# Reviewed article: Alencar JVA, Carvalho DPC, Umeno HG, França MASA, Azevedo MN. Intersectional analysis of the social vulnerability of individuals with differences in the time between oral cancer diagnosis and treatment: a cross-sectional study, Brazil, 2011-2020. Epidemiol Serv Saude. 2025:34;e20240849

**DOI:** 10.1590/S2237-96222025v34e20240849.a

**Published:** 2025-09-01

**Authors:** Cristiane Rocha Magalhães

**Affiliations:** 1 Instituto Nacional de Câncer, Rio de Janeiro, RJ, Brazil

## Peer review A

## Questionnaire

Does the manuscript contain new and significant information to justify publication? Yes

Does the Abstract (Summary) clearly and accurately describe the content of the article? Yes

Is the problem significant and concisely stated? Yes

Are the methods described comprehensively? Yes

Are the interpretations and conclusions justified by the results? Yes

Is adequate reference made to other work in the field? Yes

## Manuscript Structure

Length of article is: Adequate

Number of tables is: Adequate

Number of figures is: Adequate

Please state any conflict(s) of interest that you have in relation to the review of this paper: None

Interest: Excellent

Quality: Good

Originality: Good

Overall: Good

Recommendation: Minor Revision

## Comments

O tema é altamente relevante para a Saúde Coletiva, especialmente no contexto brasileiro, onde disparidades no acesso ao tratamento do câncer são marcantes. A abordagem interseccional é inovadora e permite compreender as desigualdades de maneira mais refinada.

A utilização do Sis-RHC/INCA é pertinente, pois é uma fonte robusta de informações sobre câncer no Brasil. No entanto, seria interessante uma discussão sobre possíveis falhas no preenchimento dos registros hospitalares, como subnotificação e diferenças regionais que podem afetar a qualidade dos dados e a precisão das estimativas.

A exclusão de uma grande parte da amostra pode comprometer a representatividade dos dados. O artigo menciona que os dados excluídos não continham todas as variáveis possíveis, mas não há uma análise sobre possíveis diferenças entre os casos incluídos e excluídos, o que pode comprometer a validade externa dos achados.

A regressão logística multinomial é uma escolha estatística coerente para avaliar a associação entre os perfis e os tempos de início do tratamento. No entanto, não está claro se foram avaliadas possíveis interações entre as variáveis independentes. Além disso, a ausência de ajustes para potenciais confundidores além do estadiamento do tumor pode ser uma limitação. Também não há informações sobre comorbidades ou outros fatores clínicos que pudessem influenciar o tempo para o início do tratamento.

A categorização do tempo até o tratamento em três grupos (0-60, 61-90 e ≥91 dias) faz sentido do ponto de vista legal (Lei 12.732/2012), mas a utilização de variáveis contínuas ou a divisão dos grupos em intervalos menores poderia fornecer informações mais detalhadas.

No manuscrito, os perfis sociodemográficos foram delineados por meio da análise de Cluster, o que permitiu identificar padrões diferentes entre os indivíduos identificados com câncer de boca no Brasil. No entanto, observe-se que apenas um dos perfis específicos (Perfil D) inclui mulheres e está concentrado na região Sudeste. Gostaria de solicitar um esclarecimento sobre a distribuição das mulheres com câncer de boca em outras regiões do país. A análise de Cluster revelou a exclusão de mulheres de muitas regiões ou houve um agrupamento específico que justifica essa distribuição? Caso existam diagnósticos de mulheres em outras

regiões, seria possível incluir essa informação na descrição dos perfis?Esse detalhamento contribuirá para uma compreensão mais ampla das desigualdades regionais e de gênero no acesso ao tratamento do câncer de boca no Brasil.

